# Supplementary material for: WordCommentsAnalyzer: A windows software tool for qualitative research
Source: F1000Res. 2018 Sep 4;7:536. Originally published 2018 May 3. [Version 2] doi: 10.12688/f1000research.14819.2 (PMC6137411; doi:10.12688/f1000research.14819.2)
Supplement: Supplementary file 3 [file f1000research-7-17614-s0002.tgz › b2a79496-4ada-47c4-9682-a99ce867836d.zip!/Eight Iranian Health Journals/Journal of Health System Research _13.docx]

© Journal of Health System Research . This work is licensed under CC BY-NC 4.0 (https://creativecommons.org/licenses/by-nc/4.0/).

Volume & Issue: دوره 11، شماره 1، 1394

Title: بررسی فاکتورهای مؤثر بر باقیمانده آنتی بیوتیک تتراسایکلین در تخم مرغ: مروری بر شواهد موجود

http://hsr.mui.ac.ir/index.php/jhsr/article/view/2037

مقدمه: آنتی‌بیوتیک‎های تتراسایکلین جهت کنترل و پیشگیری از بیماری، درمان و افزایش رشد در طیور مورد استفاده قرار می‌گیرند. استفاده از آنتی‌بیوتیک در حیوانات باعث باقیماندن دارو در مواد غذایی با منشاء حیوانی مانند تخم‌مرغ می‌شود. روش‌ها: در این مطالعه کلیدواژه‌های تتراسایکلین، باقیمانده و تخم‌مرغ در پایگاه‌های اطلاعاتی PubMed Google scholar ,Elsevier در بین سال‌های 2014-1991 مورد جستجو قرار گرفتند و داده‌های لازم اخذ شدند. یافته‌ها: فاکتور‎‎های مانند زمان انتظار، جوشاندن، سرخ کردن، سیستم نگهداری، اندازه مرغداری، منطقه جغرافیایی و فصل باعث کاهش باقیمانده دارو در تخم مرغ می‎شوند. کاهش دارو در تخم‎مرغدر اثر سرد‎سازی کم می‎باشد و در مورد منبع غذا نیاز به بررسی‎های بیشتری می‎باشد. نتیجه‌گیری: با توجه به یافته‌های این مطالعه نظارت بیشتری بر استفاده از آنتی‌بیوتیک‎ها در مرغداری‌ها و هم‌چنین آموزش همگانی در مورد نحوه پخت تخم‌مرغ باعث کاهش مواجهه انسان با آنتی‌بیوتیک‎ها خواهد شد.

Title: مروری نظام‌مند بر فراوانی آفلاتوکسینM1 در شیر و سایر فرآورده های لبنی در ایران

http://hsr.mui.ac.ir/index.php/jhsr/article/view/2152

مقدمه: آفلاتوکسین‌ها متابولیت‌های ثانویه قارچی هستند که توسط بعضی از گونه‌های قارچی مانند آسپرژیلوس فلاووس، آسپرژیلوس پارازیتیکوس و به ندرت آسپرژیلوس نومیوس تولید می‌شوند. این قارچ‌ها تقریبا در همه جا وجود دارند و در طیف وسیعی از محصولات کشاورزی مانند حبوبات، غلات، میوه خشک یافت می‌شوند. آفلاتوکسین‌ها ترکیبات بسیار سمی هستند که دارای قابلیت سرطان‌زایی، جهش‌زایی، ناقص‌الخلقه‌زایی و هم‌چنین باعث سرکوب سیستم ایمنی می‌شوند و ارگان هدف برای سمیت و سرطان‌زایی، کبد می‌باشد. کمیته اروپا و کدکس حداکثر مقدار مجاز آفلاتوکسین را در شیر و سایر محصولات لبنی 50 نانوگرم در لیتر تعیین کرده‌اند. هدف از انجام این مطالعه مرور نظام‌مند مطالعات انجام گرفته در کشور و بررسی نتایج آن‌ها از حیث ایمنی شیر و فرآورده‌های لبنی از نظر وجود و مقدار آفلاتوکسینM1 می‌باشد. روش‌ها: جهت دریافت مقالات مرتبط، ذخایر علمی الکترونیک در کشور شامل SID و هم‌چنین دربانك‌های اطلاعاتی Medline،Science Direct ،PubMed ،Elsevier ،Scopus  با استفاده از کلمات کلیدیAflatoxin M1،pasteurized milk ،UHT milk ،Raw milk ،Yoghurt،cheese و معادل فارسی آن ها از سال 2005 تا 2013 جستجو شد که از 42 مقاله مرتبط با موضوع، 23 مقاله تحقیقاتی مورد استفاده قرار گرفت. یافته‌ها: آلودگی شیر و سایر فرآورده‌های لبنی مورد بررسی، در اکثر مطالعات فراتر از استاندارد تعیین شده توسط کمیته غذایی کدکس و سازمان ملی استاندارد ایران (50 نانوگرم در لیتر) بوده است. نتیجه‌گیری: بررسی مطالعات صورت گرفته در ایران گویای این مطلب است که بیشترین توجه در میان محصولات لبنی بر شیر پاستوریزه و سپس شیر استریلیزه متمرکز بوده و بقیه محصولات سهم کمی را در مطالعات صورت گرفته، برخوردارند. در اکثر مطالعات، آلودگی بالاتر از استاندارد اتحادیه اروپا و سازمان ملی استاندارد ایران بوده است؛ بنابراین سلامت مصرف‌کنندگان خصوصا" کودکان در معرض خطر است.

Title: بررسی رابطه بین ابعاد محتوایی و ابعاد ساختاری در ساختار سازمانی بیمارستان خاتم زاهدان

http://hsr.mui.ac.ir/index.php/jhsr/article/view/1115

مقدمه: ساختار سازمانی راه و شیوه‌ای است که به‌وسیله آن فعالیت‌های سازمان تعیین، سازماندهی و هماهنگ می‌گردد.وجود ساختار سازمانی مناسب و متناسب با اهداف و راهبردهای سازمان، نقش به‌سزایی در افزایش کارایی و اثربخشی دارد. در این مطالعه، ابعاد ساختاری و محتوایی ساختار سازمانی بیمارستان خاتم زاهدان تعیین و رابطه بین آن‌ها بررسی گردیده است. روش‌ها: در این مطالعه توصیفی، نظرات تمامی مدیران سطوح مختلف بیمارستان خاتم زاهدان از طریق پرسش‌نامه اخذ شده است. پایایی پرسش‌نامه به‌وسیله آزمون آلفای کرونباخ و روایی آن از طریق تأیید خبرگان انجام شد. یافته‌های به‌دست آمده به‌وسیله نرم‌افزار SPSS تحلیل شد. یافته‌ها: ساختار بیمارستان خاتم زاهدان به شکل مکانیکی با رسمیت و پیچیدگی بالا و تمرکز پایین بود.به صورت کلی، ابعاد محتوایی با ابعاد ساختاری رابطه معنی‌داری داشتند. از بین این ابعاد، ابعاد اهداف و محیط دارای رابطه و بعد فرهنگ بدون رابطه معنی‌دار بود. در شرایط هم‌زمان، بعد اهداف بیشترین تأثیر را بر ابعاد ساختاری داشت. نتیجه‌گیری: عدم تمرکز در بیمارستان خاتم زاهدان به عنوان نکته‌ای متفاوت و مثبت در مقایسه با سایر بیمارستان‌های کشور می‌باشد. نتایج مربوط به بعد فرهنگ سازمانی نیز این نکته را تأیید می‌کند. رابطه معنی‌دار بین ابعاد محتوایی وابعاد ساختاری، مشابه مطالعات قبلی در این زمینه است. تبیین و تشریح اهداف و راهبردهای بیمارستان به پرسنل و متناسب نمودن ساختار موجود با اهداف در جهت افزایش کارایی و اثربخشی در بیمارستان پیشنهاد می‌گردد.

Title: بررسی ارتباط میان خودکارآمدی ، موانع و حمایت اجتماعی درک شده با رفتارهاي ارتقا دهنده سلامت سالمندان شهرستان دنا در سال 1391-1390

http://hsr.mui.ac.ir/index.php/jhsr/article/view/1360

مقدمه: رفتارهاي ارتقادهنده سلامت يكي از معيارهاي عمده تعيين‌کننده سلامت مي‌باشند و از طرفی این رفتارها در سالمندان، تأثير بالقوه‌اي در پيشبرد سلامتي و كيفيت زندگي آنان داشته و به همان نسبت موجب كاهش هزينه‌هاي مربوط به مراقبت‌هاي بهداشتي خواهند شد. لذا این مطالعه با هدف شناسايي تأثیر خودکارآمدی، موانع و حمایت اجتماعی درک شده در تعيين اين رفتارها در سالمندان شهرستان دنا انجام شد. روش‌ها: در مطالعه حاضر که از نوع توصیفی تحلیلی بوده تعداد 120 نفر از سالمندان( شامل 59 زن و 61 مرد) شهرستان دنا به روش نمونه‌گیری تصادفی ساده انتخاب شدند. داده‌های این مطالعه از طریق پرسش‌نامه‌ای مشتمل بر قسمت‌های متفاوت (مشخصات دموگرافیگ، رفتارهاي ارتقادهنده سلامت ،خودکارآمدی، موانع و حمایت اجتماعی درک شده از رفتارهاي ارتقادهنده سلامت) جمع‌آوری گردید. از آمار توصيفي و آزمون‌های آماری همبستگی پیرسون ، t مستقل و آنالیز رگرسیون خطی چندگانه جهت تجزیه و تحلیل داده‌ها استفاده شد. یافته‌ها: میانگین نمره رفتارهای ارتقادهنده سلامت 7/10± 5/57 بود. میان خودکارآمدی و حمایت اجتماعی درک شده با رفتارهای ارتقادهنده سلامت به طور کلی و خرده مقیاس‌های مدیریت استرس، فعالیت بدنی، روابط اجتماعی بین‌فردی و پیشگیری ارتباط مثبت معنی‌دار مشاهده شد (001/0 P<). بین موانع درک شده و رفتارهای ارتقادهنده سلامت به طور کلی و خرده مقیاس مدیریت استرس رابطه معنی‌دار معکوس دیده شد. نتیجه‌گیری: با توجه به يافته‌هاي پژوهش كه بيانگر ارتباط مستقیم خودكارآمدي و حمایت اجتماعی درک شده با انجام رفتارهاي ارتقادهنده سلامت می‌باشد، مسؤولين و مراقبين بهداشتي مي‌توانند با طرح‌ريزي برنامه‌های آموزش ارتقاسلامت، زمينه افزايش خودكارآمدي و هم‌چنین خانواده‌ها نیز از طریق فراهم آوردن حمایت‌های عاطفی لازم جهت سالمندان، زمينه افزایش حمایت اجتماعی و  به دنبال آن تغيير رفتار را برای سالمندان فراهم نمايند.

Title: تأثیر برنامه آموزش سلامت بر استرس شغلی پرستاران زن

http://hsr.mui.ac.ir/index.php/jhsr/article/view/1451

مقدمه: استرس شغلی یکی از مهم‌ترین خطرات شغلی در عصر مدرن است، به نحوی که هم برای فرد و هم سازمان موجب خسارت و زیان‌های فراوان می‌شود و پرستاری از مشاغل پر استرس است. این مطالعه با هدف تعيين تأثیر آموزش بهداشت بر کاهش استرس شغلی در پرستاران انجام شد. روش‌ها: این پژوهش نیمه تجربی درسال 89 در بيمارستان‌هاي دولتي همدان و بر روی 58 کارشناس پرستاری که به‌طور تصادفی در گروه مداخله و شاهد قرار گرفتند انجام شد.جهت سنجش استرس شغلی از مقیاس استرس شغلی پرستاران (NSS یا nursing stress scale) و جهت سنجش آگاهی، نگرش، مهارت‌ها و رفتارهای خودیاری از پرسش‌نامه محقق ساخته استفاده شد. برنامه آموزشی طی 5 جلسه به مدت 3 هفته برای گروه مداخله انجام شد و سپس پرستاران به مدت 5/1 ماه پیگیری شدند. برای تحلیل داده‌ها از نرم‌افزار spss13 وآزمون آماری t تست، مان ویتنی و ویل کاکسون استفاده شد. یافته‌ها: میانگین نمره استرس شغلی قبل از آموزش در 2 گروه اختلاف معنی‌دار نداشت. اما بعد از آموزش میانگین نمره استرس شغلی در گروه مداخله نسبت به شاهد کاهش معنی‌دار داشت (001/0 p<) هم‌چنین میانگین نمرات آگاهی، نگرش، مهارت‌ها و رفتارهای خودیاری پس از آموزش در گروه مداخله افزایش معنی‌دار داشت (001/0 p<). نتیجه‌گیری: یافته‌ها تأثیر برنامه آموزش بهداشت را در افزایش رفتارهای خودیاری و کاهش استرس شغلی پرستاران تأیید می‌کنند.

Title: بررسی پیشگویی‌کننده‌های رفتارهای پیشگیری‌کننده ازپوکی استخوان در زنان شهر فسا:تلفیقی ازمدل اعتقاد بهداشتی وتئوری شناختی اجتماعی

http://hsr.mui.ac.ir/index.php/jhsr/article/view/1793

مقدمه: بررسي عوامل مؤثر بر اتخاذ رفتارهای پيشگيري‌کننده ازپوکی استخوان در زنان با استفاده از الگوهايي كه عوامل مؤثر بر رفتار را شناسايي و تقويت مي‌كنند ضروري مي‌باشد. پژوهش حاضر با هدف تعیین رفتارهای پیشگیری‌کننده ازپوکی استخوان در زنان شهر فسا با استفاده ازمدل اعتقاد بهداشتی و تئوری شناختی اجتماعی درسال 1392 انجام شد. روش‌ها: در اين مطالعه مقطعي تحليلي 401 نفر از زنان 30 تا 50 سال تحت پوشش مراکز بهداشتی درمانی شهر فسا به صورت تصادفی انتخاب شدند. مقياس مربوط به عوامل مدل اعتقاد بهداشتي (حساسيت، شدت، تهديد، منافع، موانع و خودكارامدي درك شده وراهنماهاي عمل) و سازه‌های خودتنظیمی و حمایت اجتماعی از تئوری شناختی اجتماعی و عملكرد تغذیه‌ای و پیاده‌روی جهت پیشگیری از پوکی استخوان در زنان سنجيده شد. در نهايت اطلاعات با استفاده نرم‌افزار SPSS نسخه 16 و از طریق آزمون‌های آماری توصیفی (میانگین و انحراف معیار) وآزمون‌هاي تحليلي شامل ضريب همبستگي پيرسون، آنالیز رگرسیون چند متغیره خطی ،آناليز واريانس يك‌طرفه و آزمون تي مستقل تجزيه و تحليل شد و سطح معنی‌داری کمتر 05/ در نظر گرفته شد. یافته‌ها: میانگین سنی زنان مورد مطالعه 2/6±9/40 سال بود. متغیرهای حساسیت درک شده، انگیزش، حمایت اجتماعی و خودتنظیمی برای رفتار پیاده‌روی ومتغیرهای حساسیت درک شده و خودتنظیمی برای رفتار تغذیه‌ای پیشگویی‌کننده بود. بین عملکرد پیاده‌روی و حساسیت درک شده (136/0=r و007/0=p)، انگیزش (120/0=r و016/0=p)، حمایت اجتماعی (068/0=r و030/0=p) و خودتنظیمی (118/0=r و018/0=p) ارتباط معنی‌داری وجود داشت. در این بررسی بین عملکرد تغذیه‌ای و حساسیت درک شده (068/0=r و003/0=p)، خودکارآمدی (039/0=r و042/0=p) و خودتنظیمی (069/0=r و070/0=p) ارتباط مستقیم و با موانع درک شده (047/0- =r و050/0=p) ارتباط معکوس معنی‌داری وجود داشت. متغیرهای مورد بررسی 1/29 درصد از واریانس رفتار پیاده‌روی و 2/20 درصد از واریانس رفتار تغذیه‌ای پیشگیری از پوکی استخوان را بیان نمودند. نتیجه‌گیری: مطالعه حاضر كارايي الگوي اعتقاد بهداشتي و سازه‌های خودتنظیمی و حمایت اجتماعی را در پيشگويي اتخاذ رفتارهای تغذیه‌ای و پیاده‌روی جهت پیشگیری از پوکی استخوان نشان داد. از اين رو اين الگوها مي‌تواند به عنوان چارچوبي جهت طراحي و اجراي مداخلات آموزشي جهت پیشگیری از پوکی استخوان در زنان استفاده شود و به ارتقا و حفظ سلامت آنان كمك نمايد.

Title: نگرش‏های ناکارآمد و ارتباط آن با استرس، اضطراب و افسردگی در مبتلایان به سرطان‏ پستان

http://hsr.mui.ac.ir/index.php/jhsr/article/view/1795

مقدمه: سرطان پستان به عنوان شايع‏ترين و تأثیرگذارترين سرطان از نظر عاطفي و رواني در بین زنان، موجب بروز مشكلات هيجاني و عاطفي عميقي همچون استرس، اضطراب و افسردگی در بيمار و خانواده وي مي‏شود. یکی از عوامل احتمالي موثر در پیدایش افسردگی، اضطراب و استرس در افراد بر اساس مدل‌های شناختی، تحریف‌های شناختی و نگرش‏های ناکارآمد نسبت به شرایط است. شيوع بالا و تاثیرات گسترده و مهم استرس، ‌اضطراب و افسردگي در بيماران مبتلا به سرطان پستان در ابعاد مختلف زندگی بیماران و تأثیر نگرش‏های ناکارآمد در شکل‏گیری این مؤلفه‏ها، محققان را برآن داشت تا مطالعه‏اي را با هدف تعيين ارتباط بين نگرش‏هاي ناكارآمد با استرس، ‌اضطراب و افسردگي در مبتلایان به سرطان پستان اجرا نمایند. روش‌ها: این مطالعه، پژوهشی از نوع توصیفی همبستگی بود که به شکل مقطعی اجرا شد. 297 بیمار مبتلا به سرطان پستان مراجعه کننده به یکی از مراکز درمانی تخصصی سرطان اصفهان، به روش در دسترس در مدت 6 ماه وارد مطالعه گردیدند. برای جمع‌آوری اطلاعات از چك ليست اطلاعات دموگرافیک و اطلاعات موجود پرونده بیمار، پرسشنامه سنجش نگرش‏های ناکارآمد (DAS-26 یا dysfunctional attitudes questionnaire) و پرسش‌نامه سنجش استرس، اضطراب و افسردگی (DASS-42 یا depression, anxiety, and stress scale) استفاده شد. داده‌ها با استفاده از نرم افزار آماری SPSS18 و بکارگیری روش‌های آمار توصیفی و تحلیلی، مورد تجزیه تحلیل قرار گرفت. یافته‌ها: میانگین نمره نگرش‏های ناکارآمد واحدهای مورد پژوهش 35/87 (53/27) بود. همچنین میانگین نمره استرس، اضطراب و افسردگی واحدهای پژوهش به ترتیب 16/14 (38/9)، 64/11 (27/8) و 06/12 (52/9) بود. نتايج نشان داد كه ارتباط آماری معنی‏داری بین نگرش‏های ناکارآمد با استرس، اضطراب و افسردگی در بیماران مبتلا به سرطان پستان وجود دارد (05/0p<). نتیجه‌گیری: بر اساس يافته‌هاي مطالعه‌ حاضر در مبتلايان به سرطان پستان با نمرات بالاتر نگرش‏هاي ناكارآمد، ميزان استرس، اضطراب و افسردگي بيشتري وجود داشت. به علاوه پايين بودن نمره نگرش‏هاي ناكارآمد يك فاكتور مهم محافظت كننده در برابر بروز استرس، اضطراب و افسردگي در اين بيماران مي باشد.

Title: بررسی ارتباط بین نوبت کاری و فرسودگی شغلی در کارمندان پرستار بیمارستان الزهرا با کاربرد پرسش‌نامه فرسودگی مسلش

http://hsr.mui.ac.ir/index.php/jhsr/article/view/1836

مقدمه: فرسودگی شغلی بر سلامت جسمی و روحی اثرگذار بوده و به‌عنوان پیامد نهایی استرس شغلی در بسیاری از مشاغل از جمله پرستاری مشاهده می‌شود.هدف از این مطالعه بررسی ارتباط نوبت کاری و فرسودگی شغلی در بخش‌های مختلف بیمارستان الزهرا اصفهان بود. استفاده از نتایج این مطالعه در برنامه‌ریزی‌های بهداشتی- درمانی می‌تواند موجب کاهش فرسودگی شغلی و در نتیجه افزایش کیفیت خدمات و مراقبت‌های بهداشتی، افزایش رضایت بیمار، کاهش غیبت از کار، افزایش اثربخشی هزینه‌ها و بهره‌وری گردد. روش‌ها: این مطالعه از نوع مطالعات توصیفی– تحلیلی بوده و جمعیت مورد مطالعه پرستاران روزکار و شیفتی بیمارستان الزهرا اصفهان بودند که نمونه‌گیری به روش طبقه‌ای صورت گرفت. داده‌های مورد نیاز توسط دو پرسش‌نامه عمومی شامل سؤالاتی در خصوص متغیرهای زمینه‌ای گروه هدف و پرسش‌نامه تخصصی فرسودگی شغلی مسلش (Maslach Burnout Inventory) جمع‌آوری شد، بدین منظور در شیفت‌های مختلف کاری به بیمارستان مراجعه و ضمن توضیح در خصوص اهداف مطالعه و آموزش دستورالعمل و نحوه تکمیل، پرسش‌نامه در اختیار پرستارانی که همکاری خود را اعلام کرده بودند قرار گرفت. یافته‌ها: در مقایسه میزان فرسودگی شغلی پرستاران در شیفت‌های مختلف، بیشترین فرسودگی شغلی در بعد خستگی هیجانی و مسخ شخصیت در شیفت عصر و کفایت شخصی در شیفت شب بود. بیشترین امتیاز فرسودگی شغلی در بعد خستگی هیجانی در بخش عروق، مسخ شخصیت در بخش دیالیز و کمترین امتیاز در بعد کفایت شخصی در بخش ICU دیده شد. این پژوهش نشان داد که ارتباط شیفت با متغیر‌‌های وابسته در بخش‌های مختلف متفاوت است (001/0 P <). نتیجه‌گیری: وجود سطوح بالای خستگی هیجانی و مسخ شخصیت سطح پایین بعد کاهش احساس کفایت شخصی در پرستاران مورد مطالعه می‌تواند هشداری برای مدیران مربوطه تلقی شود، چرا که در صورت عدم مدیریت مناسب برای مهار آن، می‌تواند منجر به آسیب‌های فراوانی بر نظام سلامت شود.

Title: میزان انطباق واحدهای مامایی و وازکتومی مرکز بهداشت شماره دو اصفهان با معیارهای مطلوب کنترل عفونت این مرکز و عوامل مرتبط با آن

http://hsr.mui.ac.ir/index.php/jhsr/article/view/1842

مقدمه: پرسنل بهداشتي به خصوص ماماها و پزشکان با رعايت معیارهای کنترل عفونت مي‌توانند خطر بروز عفونت‌هاي پس از جايگذاري آی یو دی (IUD) یا انجام وازکتومی و انتقال بيماري‌هايي مثل هپاتيت و ايدز را كاهش دهند. با توجه به آمار قابل توجه IUD و وازکتومی و وجود مشکل در زمینه کنترل عفونت در واحدهای بهداشتی و خلا تحقیقی موجود در واحدهای مامایی در بررسی متون؛ این پژوهش به منظور تعیین وضعیت کنترل عفونت در واحدهای مامایی و وازکتومی و میزان انطباق آن با معیارهای کنترل عفونت این مرکز انجام گرفت. روش‌ها: پژوهش حاضر مطالعه‌ای توصیفی تحلیلی در سال 1391 و نمونه‌های پژوهش 78 نفر پرسنل ارایه دهنده‌ی خدمت شامل پزشک، کارشناس و کاردان مامایی در65 واحد مامایی و وازکتومی بودند.ابزار جمع‌آوری اطلاعات، یک پرسش‌نامه محقق ساخته مربوط به کارکنان (مشخصات دموگرافیک پرسنل و محل کار– معیارهای حفاظت شخصی و حفاظت گیرنده خدمت) و یک فهرست وارسی مربوط به محیط واحدهای مورد پژوهش (مشخصات دموگرافیک و معیارهای دفع مواد زائد عفونی، محیط کار، شستشو و ضدعفونی و استریلیزاسیون وسایل) بود.برخی از شاخص‌ها از نظر ضرورت انجام، باید انجام می‌گرفت (Must to do) .برحسب این که فرد یا واحد ارائه‌دهنده خدمت دارای چند درصد از معیارهای مطلوبیت کنترل عفونت بودند، رتبه آن‌ها به ضعیف (59-0%)، متوسط (79-60%) و خوب (80%به بالا) تقسیم گردید.معیار مورد قبول، کسب حداقل 75 % امتیاز معیارهای کنترل عفونت و حداقل 80 % از امتیازهای « Must to do » در نمونه‌های مورد پژوهش بود. داده‌های حاصله در نرم‌افزار  SPSS19و روش‌های ماری توصیفی و تحلیلی و آزمون کای دو و سطح اطمینان 95% مورد بررسی و تجزیه و تحلیل قرار گرفت. یافته‌ها: افراد و واحدهای مورد پژوهش از نظر میزان انطباق با معیارهای کنترل عفونت این مرکز رتبه‌بندی شدند. پرسنل: 8/12% (10N=) ضعیف، 3/60 % (47 N=) متوسط و 9/26% (21 N=) خوب و واحدها: 2/35% (19 N=) ضعیف، 7/53% درصد (29 N=) متوسط و 11/1 درصد (6 N=) خوب بودند.5/11% پرسنل(9N=) و 01/7 درصد واحدها (4N=) از نظر میزان انطباق با معیارهای مطلوبیت کنترل عفونت این مرکز (75% نمره و 80% بایدها) شرایط مطلوب داشتند. نتیجه‌گیری: افراد و واحدهای مورد پژوهش از نظر میزان انطباق با معیارهای کنترل عفونت این مرکز رتبه‌بندی شدند. پرسنل: 8/12% (10N=) ضعیف، 3/60 % (47 N=) متوسط و 9/26% (21 N=) خوب و واحدها: 2/35% (19 N=) ضعیف، 7/53% درصد (29 N=) متوسط و 11/1 درصد (6 N=) خوب بودند.5/11% پرسنل(9N=) و 01/7 درصد واحدها (4N=) از نظر میزان انطباق با معیارهای مطلوبیت کنترل عفونت این مرکز (75% نمره و 80% بایدها) شرایط مطلوب داشتند.میزان انطباق واحدهای مامایی و وازکتومی مرکز بهداشت شماره دو اصفهان با معیارهای کنترل عفونت، به جز در موارد انجام واکسیناسیون هپاتیت B و جمع‌آوری زباله‌های زائد عفونی در سایر موارد، مطلوب نمی‌باشد. لذا به نظر می‌رسد آموزش و پایش مستمر کارکنان، هم‌چنین خرید مواد ضدعفونی استاندارد، باید در اولویت برنامه‌های مرکز بهداشت قرار گیرد.

Title: بررسی میزان شیوع غیبت از کار و عوامل مؤثر برآن در یکی از صنایع استان مازندران در سال‌های 1386 تا 1391

http://hsr.mui.ac.ir/index.php/jhsr/article/view/1892

مقدمه: غیبت از کار، می‌تواند به‌طور جدی تأثیر منفی بر کسب و کار داشته باشد. این مطالعه جهت ارزیابی وضعیت سلامت شغلی کارکنان این صنعت براساس شاخص‌های غیبت و عوامل مؤثر بر آن صورت گرفت. روش‌ها: این مطالعه از نوع طولي بود. 128پرونده حادثه منجر به غیبت در سال‌های 86 تا 91 مورد بررسی قرار گرفت. مطالعه غیبت ناشی از کار، براساس شاخص‌های  نرخ زمان از دست رفته، میزان کل موارد غیبت، میزان موارد غیبت به علت بیماری، میزان فرکانس غیبت، میانگین تعداد روزهای غیبت به ازاء هر فرد و فاکتور برادفورد محاسبه شد. پس از جمع‌آوری داده‌ها، به منظور تجزیه و تحلیل نتایج از نرم‌افزار SPSS نسخه 20 و آزمون‌های مربع کای و آنالیز واریانس و نسبت شانس استفاده شد. یافته‌ها: نرخ زمان ازدست رفته،  میزان کل موارد غیبت، میزان موارد غیبت به علت بیماری،مجموع کل روزهای غیبت از کار غیرموجه و میانگین تعداد روزهای غیبت به ازاء هر فرد به ترتیب 956/0، 055/1، 726/0، 3265/0 و 41/216% بود. هم‌چنین فاکتور تجمعی برادفورد در سال های 89 ، 90 و 91 به ترتیب 86 ، 19 و 71 به دست آمد. براساس نتایج آزمون‌های آماری، مشخص شد که بین عامل وقوع حادثه و غیبت (032/0 = P)، ماه وقوع و غیبت (048/0 P=)، ماهیت آسیب‌دیدگی و غیبت از کار (016/0P=)، شیفت کاری و فاکتور برادفورد (04/0 P=)، فاکتور برادفورد و عامل حادثه (032/0 P=) و حقوق دریافتی و زمان وقوع حادثه (024/0 P=) ارتباط معنی‌داری وجود داشت. نتیجه‌گیری: براساس نتایج این مطالعه، آمار غیبت از کار در سال 91 بیشتر از سال‌های قبل بود که بایستی علت‌یابی و کنترل شود. هم‌چنین مشخص شد که موارد مختلفی می‌تواند بر روی میزان غیبت ناشی از کار مؤثر باشد، از جمله این عوامل می‌توان به سن افراد، نوبت کاری، وضعیت استخدامی افراد، نوع حادثه، ماه‌های سال و … اشاره کرد. با بررسی و علت‌یابی غیبت ناشی از کار، می‌توان آمار آن را در سازمان کاهش داد و در نتیجه آن بهره‌وری و تولید را افزایش داد.

Title: امکان سنجی استفاده از فرایند اکسیداسیون پیشرفته (O3/H2O2) بر روی تأثیر داروی دیکلوفناک بر فعالیت متان سازی ویژه جرم زیستی بیهوازی

http://hsr.mui.ac.ir/index.php/jhsr/article/view/1899

مقدمه: دفع ترکیبات دارویی به محیط زیست به عنوان آلاینده‌های نو ظهور، نگرانی قابل توجهی ایجاد کرده و استفاده از روش‌های جدید تصفیه فاضلاب جهت حذف این ترکیبات ضروری به نظر می‌رسد. هدف از مطالعه حاضر امکان‌سنجی استفاده از فرایند ازناسیون بر روی تأثیر داروی دیکلوفناک بر فعالیت متان‌سازی ویژه جرم زیستی بیهوازی است. روش‌ها: در این مطالعه تعداد 12 آزمایش هضم بیهوازی به منظور بررسی میزان بازدارندگی داروی دیکلوفناک بر فعالیت متان‌سازی ویژه جرم زیستی بیهوازی به روش ناپیوسته قبل و پس از کاربرد فرایند اکسیداسیون پیشرفته (O3/H2O2) در راکتورهایml  500 که 30% جرم زیستی بیهوازی و 70% سوبستره بود، انجام شد. تکنیک مورد استفاده در این مطالعه جهت سنجش متان تولیدی روش جابجایی مایع بود. مدت هر آزمایش در حدود 8 تا 16روز به طول انجامید. یافته‌ها: میزان متان تجمعی در غلظت‌های 25، 50، 100، 175، 250 و mg/l350 داروی دیکلوفناک به‌ترتیب 24/764، 42/845، 82، 09/102،42/25 و ml64/1اندازه‌گیری شد. این میزان با کاربرد فرایند پیش تصفیه O3/H2O2 به مدت 180 دقیقه در غلظت‌های 25، 100، 250 و  mg/l350 به ترتیب 61/8، 65/26، 1/4 و ml1/4 مشاهده شد که نشان می‌دهد کاربرد فرایند ازن نه تنها سبب افزایش تولید متان نشد بلکه در تمامی غلظت‌ها تولید متان پس از فرایند ازن کاهش پیدا کرد. نتیجه‌گیری: داروی دیکلوفناک در غلظت‌های بالا بر روی فعالیت هاضم‌های بیهوازی اثر بازدارندگی شدیدی دارد و هم‌چنین در اثر تجزیه این دارو پس از روش پیش تصفیه با فرایند ازناسیون احتمالاً ترکیبات واسطه‌ای ایجاد شده که اثرات شدیدتری بر باکتری‌های بیهوازی در هاضم‌ها داشته و قابلیت تجزیه بیولوژیکی کمتری دارند.

Title: بررسی وظایف و آثار مدیریت ارتباط با مشتری در بیمارستان‌های منتخب شهر اصفهان

http://hsr.mui.ac.ir/index.php/jhsr/article/view/1990

مقدمه: آن‌چه که امروز برای سیستم بهداشت و درمان ارزش می‌آفریند، ایجاد ارتباط مستمر با مشتری است و سازمان‌های موفق تنها سازمان‌هایی هستند که قادر هستند، مشتریان تمام عمر برای خود ایجاد کنند و رسالت پاسخ‌گویی به مشتریان را در کمترین زمان، با بهترین کیفیت و کمترین هزینه به انجام برسانند. یکی از استراتژی‌هایی که به این سازمان‌ها کمک می‌کند، ایجاد ارتباط مستمر با مشتری است. روش‌ها: اين پژوهش از نوع تحقيقات توصیفی– تحلیلی است. جامعه پژوهش پرستاران بيمارستان‌های منتخب شهر اصفهان بودند که نمونه‌گیری به روش تصادفی طبقه‌ای صورت گرفته است. ابزار جمع‌آوري داده‌ها پرسش‌نامه محقق ساخته‌ی مدیریت ارتباط با مشتری بوده که اعتبار و روايي آن مورد تأييد قرار گرفته است. برای تعیین عوامل از تحلیل عاملی تأییدی استفاده شد. روش تحلیل داده‌ها،مدل معادلات ساختاری بوده و نرم‌افزارهای مورد استفاده (18) SPSS و Amos(18) بوده است. یافته‌ها: در بعد وظایف مدیریت ارتباط با مشتری تنوع خدمات دارای بیشترین امتیاز (9/2) و در آثار مدیریت ارتباط با مشتری بهره‌وری دارای بیشترین امتیاز (2/3) می‌باشد. در بین ابعاد مدل نهایی بهره‌وری دارای بیشترین میزان تأثیر و تعامل با مشتری و حفظ مشتری دارای کمترین میزان تأثیر می‌باشند. نتیجه‌گیری: سازمان‌های خدمات بهداشتی و درمانی با پياده‌سازي مديريت ارتباط با مشتري می‌توانند در بازار رقابت باقی بمانند. بيمارستان‌هاي مورد مطالعه لازم است استراتژي‌هاي كسب اطلاعات، رضايت‌سنجي و وفاداري در مورد مشتريان، جذب و نگهداشتن مشتریان جديد را ارتقا ببخشند.

Title: حذف سرب از پساب فاضلاب سنتتیک صنایع باتری سازی توسط نانوذرات مگهمایت ( Fe2O3-γ)

http://hsr.mui.ac.ir/index.php/jhsr/article/view/2196

مقدمه: سرب یکی از آلاینده‌های سمی اولویت‌دار محسوب می‌شود و از پساب صنایعی از قبیل باتری‌سازی، رنگ‌كاري،ساخت تسليحات، شيشه‌سازي، صنعت چاپ و غیره وارد محیط زیست می‌گردد. در این تحقیق نانوذرات اکسید آهن مغناطیسی (مگهمایت Fe2O3-g) به منظور ، حذف سرب از پساب سنتتیک صنایع باتری‌سازی مورد بررسی قرار گرفت. روش‌ها: دراین مطالعه تأثیر عوامل مختلف شامل pH، غلظت اولیه سرب و زمان تماس بر حذف سرب از پساب سنتتیک توسط نانو ذرات مگهمایت مورد بررسی قرار گرفت و پس از جداسازی جاذب توسط میدان مغناطیسی، سنجش غلظت سرب باقیمانده در مایع رویی با دستگاه جذب اتمی مدل پرکین المر 2380 صورت گرفت. سپس نتایج توسط نرم افزار SPSS نسخه18 آنالیز گردید. یافته‌ها: نتایج این تحقیق نشان دادکه در شرایط pH معادل 7، غلظت اولیه سرب 50 میلی‌گرم بر لیتر و نانو مگهمایت با دوز 1/0 گرم بر لیتر، زمان تماس 40 دقیقه، سرعت اختلاط 150 دور بر دقیقه، راندمان حذف سرب 91/63% و ظرفیت جذب نیز mg/g61 است. نتیجه‌گیری: در شرایط بهینه نانوذرات مگهمایت ظرفیت جذب بالا، سریع و مؤثر فلز سرب را داشته و راندمان حذف با pH و زمان رابطه مستقیم و با غلظت اولیه سرب رابطه عکس داشته و مدل جذب با ایزوترم فروندلیچ مطابقت دارد.

Title: تعیین عوامل مؤثر بر رفتار باروری در شهرستان شهرکرد سال 1389

http://hsr.mui.ac.ir/index.php/jhsr/article/view/1533

مقدمه: اهمیت باروری به عنوان مهم‌ترین عامل تغییر در ساخت و اندازه جمعیت موجب شده تا مطالعات مربوط به باروری و عوامل تأثیرگذارنده بر آن، جایگاه ویژه‌ای را در مطالعات جمعیت‌شناسی به خود اختصاص داده است. از آن‌جا که پژوهش در جنبه‌های مختلف جمعیتی همانند مرگ و میر، باروری و مهاجرت می‌تواند گامی مؤثری در راستای طراحی و تدوین برنامه‌ها و سیاست‌های جمعیتی باشد، این مطالعه با هدف تعیین عوامل مؤثر بر تفاوت باروری در بین اقوام مختلف شهرستان شهرکرد انجام شد. روش‌ها: در این مطالعه توصیفی و پیمایشی266 نفر از زنان شوهردار 15 تا 49 سال شهرستان شهرکرد به روش نمونه‌گیری تصادفی طبقه‌بندی شده مورد بررسی قرار گرفتند. ابزار گردآوری پرسش‌نامه‌ای شامل 4 حیطه اقتصادی، اجتماعی، رفتار باروری و فرهنگی و قومی بود. پرسش‌های با طیف لیکرت از نمره 1 تا 5 نمره‌گذاری شد. داده‌های به دست آمده با نرم افزار  SPSSبا استفاده از روش‌های آمار توصیفی، آزمون استقلال آماری، آنالیز واریانس، ضریب توافق kappa و مدل خطی تعمیم یافته تجزیه و تحلیل گردید. یافته‌ها: در عوامل اجتماعی بین سن ازدواج و تعداد فرزندان تأثیر معنی‌داری وجود داشت (000/0=P). در عوامل فرهنگی متغیر تحصیلات (012/0=P) و وابستگی ایلی (005/0=P) در رفتار باروری تأثیر معنی‌داری داشت. در عوامل اقتصادی بین متغیرهای شغل شوهر (020/0=P) و مالکیت محل سکونت (022/0=P) با تفاوت رفتار باروری رابطه معنی‌دار وجود داشت. نتایج نشان داد توافق نسبی بین پاسخگویان و شوهرانشان در مورد تعداد فرزندان دلخواه وجود داشت (547/0=K)، در مورد تکرار باروری با اختلاف بسیار کمی معنی‌دار نبود (05/0P>). بین عامل فرهنگی قومی و رفتار باروری رابطه معنی‌دار وجود داشت، این رابطة سیستماتیک به صورت غیرخطی و با شدت 093/0 بود (000/0=P). نتیجه‌گیری: در بین زمینه‌های مورد مطالعه عوامل فرهنگی‌ـ قومی بیشترین تأثیر را بر رفتار باروری داشت. سطح تحصیلات و وابستگی ایلی در بین زنان اقوام مختلف نیز مؤثر بود. از بین عوامل اجتماعی متغیر سن ازدواج تأثیر معنی‌داری بر رفتار باروری داشت. آموزش زنان جهت ارتقاء سطح آگاهی آنان می‌تواند گامی مؤثر در تغییر نگرش افراد در مورد باروری باشد.

Title: شناسایی عوامل مؤثر بر فواصل زمانی میان اهداهای خون با بکارگیری مدل شکنندگی مشترک کاکس به روش بیزی: یک مطالعه مقطعی بر حسب نمونه‌ای از افراد مراجعه‌کننده به مراکز انتقال خون در ایران

http://hsr.mui.ac.ir/index.php/jhsr/article/view/2169

مقدمه: شناسایی افراد مستعد اهدای خون نقش زیادی در تهیه خون سالم دارد. هرچه فاصله زمانی بین دو اهدای خون یک داوطلب اهدای خون کمتر باشد، شانس این‌که داوطلب اهدای خون به یک فرد اهداکننده مستمر خون تبدیل شود افزایش می‌یابد. هدف از انجام این مطالعه، شناسایی عوامل مؤثر بر فواصل زمانی بین اهداهای خون در یک نمونه از اهداکنندگان بار اول خون است. روش‌ها: در مطالعه‌ای طولی از اطلاعات 864 نفر اهداکننده خون در پایگاه انتقال خون شهرکرد استفاده شد. با تنظیم یک پرسش‌نامه، اطلاعات مربوط به سن، جنس، وزن، وضعیت تأهل، محل سکونت، سطح سواد، شغل، تعداد دفعات اهدا و فاصله زمانی بین دو اهدای خون در مقیاس روز جمع‌آوری شد. از نظر چارچوب داده‌های بقا، فواصل زمانی بین اهداهای خون جزء پیشامدهای بازگشتی محسوب می‌شوند. برای شناسایی عوامل خطر موثر بر فاصله‌های زمانی بین داده‌های بقای بازگشتی، با توجه به همبستگی زمان‌های بقا برای هر فرد، مدل کاکس با وجود اثر شکنندگی مشترک گاما به داده‌ها برازش داده شد و برآورد پارامترها تحت رویکرد بیزی و بر اساس الگوریتم‌های مونت کارلوی زنجیر مارکوفی و به کمک نرم افزار WinBUGS به دست آمد. یافته‌ها: مدل خطر متناسب کاکس با وجود اثر شکنندگی مشترک گاما در مقایسه با مدل خطر متناسب کاکس بدون اثر شکنندگی برازش بهتری را بر روی داده‌های بقای بازگشتی اهدای خون نشان داد. بر اساس نتایج مدل کاکس با شکنندگی گاما، متغیر وزن بطور معنی‌داری شانس اهدای خون را افزایش و فاصله زمانی بین اهداهای خون را کاهش داده است. هم‌چنین متغیر سن به‌طور جزیی تأثیر منفی بر شانس اهدای خون داشته است به‌طوری‌که شانس اهدای خون برای افرادی که سن کمتری داشته‌اند بیشتر و فواصل زمانی بین اهداهای خون آن‌ها کمتر بوده است. نتیجه‌گیری: با توجه به برازش بهتر مدل شکنندگی مشترک کاکس بر داده‌ها، همبستگی در بین زمان‌های بازگشتی اهدای خون هر فرد تأیید می‌گردد. نظر به این‌که فاصله بین اهداهای خون برای افراد با وزن کمتر و سن بالاتر بیشتر بوده است لذا لازم است با فرهنگ‌سازی و افزایش آگاهی این افراد را به اهدای خون در فواصل زمانی کوتاه‌تر ترغیب نمود.

Title: بررسی میانگین فیتات درانواع نان درشهرستان اصفهان درسال 1385

http://hsr.mui.ac.ir/index.php/jhsr/article/view/1881

مقدمه: نان عمده‌‌ترین غذا در تأمین انرژی دریافتی روزانه در ایران می‎‎باشد. فیتات ترکیب موجود در نان است که بامواد معدنی ایجاد رسوب می‎کند و این عناصر را در دستگاه گوارش غیر قابل جذب می‎کند. این مطالعه با هدف بررسی مقدار فیتات در انواع نان در شهر اصفهان صورت گرفته است. روش‌ها: نان‎های مورد آزمایش از 6 نوع نان شامل نان کامل (سبوس دار)، نان سنگک، نان بربری، نان کمباینی و نان تافتون و نان ماشینی بودند. نمونه های نان از نانوایی‎های سطح شهر اصفهان به صورت نمونه‎گیری تصادفی تهیه شدند.مقدار فیتات به روشEarly & Beturk  اندازه‌گیری شد. یافته‌ها: نان سنگک، سبوس‌دار، کمباینی، تنوری ، بربری و ماشینی  به ترتیب بیشترین تا کمترین مقدار مقدار فیتات را داشتند. نتیجه‌گیری: درصد سبوس در آرد مصرف شده برای پخت نان، مقدار مصرف خمیر مایه، مقدار جوش شیرین اضافه شده به خمیر و مدت زمان استراحت خمیر مواردی هستند که در میزان باقی ماندن اسید فیتات در نان پس از پخت تأثیر گذارند.

Title: تحلیل فضایی اثرات عوامل محیطی و اقلیمی بر بیماری سرطان پوست در استان اصفهان

http://hsr.mui.ac.ir/index.php/jhsr/article/view/2140

مقدمه: تحقیقات جغرافیایی سلامت، اثر عوامل محیطی را بر تندرستی انسان‎ها بررسی می‎کند. محيط زيست انساني و طبيعي به ميزان وسيعي از شرايط اقلیمی تأثير مي‌پذيرد. سرطان‌هاي پوست در اکثر کشورهاي دنيا از جمله ایران در رديف شايع‌ترين‌ها هستند و طبق مطالعات انجام شده متأثر از برخی عوامل محیطی می‌باشند. هدف پژوهش حاضر تحلیل اثر عوامل اقلیمی و محیطی بر وقوع سرطان پوست در استان اصفهان می‌باشد. روش‌ها: در این تحقیق از اطلاعات داده‌های اقلیمی و بیماران سرطانی 11 شهر طی یک دوره 7 ساله (1390-1384) در استان اصفهان و از روش‌هایی مانند تحلیل فراوانی، پهنه‌بندی مقادیر، همپوشانی متغیرها، تحلیل همبستگی استفاده گردید. یافته‌ها: تعداد کل مبتلایان در شهرهای مورد مطالعه جمعا 4104 نفر که 1507 نفر (72/36 درصد) زن و 2597 نفر (27/63 درصد) مرد می‌باشند. تحلیل فراوانی بیماری در استان اصفهان به تفکیک جنس و سن نشان داد در تمامی سال‌های مورد مطالعه تعداد بیماران مرد بیش از زنان و بیشترین درصد ابتلا در سن‌های بالای50 سال می‌باشد. پهنه‌بندی بیماری نیز نشان داد بخش مرکزی استان کانون بحرانی بیماری سرطان پوست می‌باشد. نتیجه‌گیری: نتایج تحلیل همبستگی به خاطر دوره آماری کوتاه بیماری (7ساله) نتوانست مبنای قابل اعتمادی برای نتیجه‌گیری با اطمینان بالا باشد. نتایج تحلیل همبستگی و همپوشانی متغیرها نشان داد بیماری و وقوع سرطان پوست با متغیرهای اقلیمی (دما (میانگین و حداکثر مطلق)، حداقل نم نسبی، بارش، ساعات آفتابی، سرعت باد) و توپوگرافی در اکثر موارد ارتباط معنی‌داری وجود ندارد و ریشه وقوع بیماری را در دیگر عوامل اقلیمی همچون تابش خورشیدی و اشعه ماوراء بنفش و سایر عوامل مؤثر بر شیوع این بیماری از جمله ژنتیک و ... باید جستجو نمود.

Title: بررسی عوامل مؤثر بر استرس شغلی در پرسنل آتش نشانی (یک مطالعه مقطعی)

http://hsr.mui.ac.ir/index.php/jhsr/article/view/1918

مقدمه: استرس شغلی یکی از پدیده‌های مهم در زندگی اجتماعی و تهدیدی جدی برای سلامتی نیروی کار می‌باشد که  هر گاه بین نیازمندی‌های شغلی و توانایی‌های کارگر تعادلی وجود نداشته باشد ایجاد می‌گردد. هدف مطالعه حاضر بررسی عوامل موثر بر استرس شغلی در پرسنل آتش نشانی بود. روش‌ها: مطالعه توصیفی- تحلیلی - مقطعی حاضر به روش سرشماری در50 نفر از کارکنان سازمان آتش نشانی شهر سنندج انجام شد. پس از جمع‎آوری اطلاعات پایه، به منظور بررسی عوامل موثر و مرتبط با استرس از پرسشنامۀ استرس شغلی موسسه بهداشت روانی آمریکا شامل 57 سوال استفاده گردید که شامل 3 بخش روابط بین فردی، شرایط فیزیکی و محیط کار فرد و علاقه مندی به کار بود. داده‌های جمع‌آوری شده با استفاده از نرم افزار 19 SPSS آنالیز گردید. یافته‌ها: 86% از کارکنان مورد مطالعه متأهل و 14% مجرد بودند. 16%کمتر از 30 سال، 52% بین 30 تا 40 سال و 32% بیشتر از 40 سال سن داشتند. 22% کمتر از 5 سال، 28% بین 5 تا10 سال، 30% بین 10 تا 15 سال و 20% بیشتر از 15 سال سابقه کار داشتند.  میانگین نمره کلی استرس در پرسنل مورد مطالعه 7/172 و در سطح  بالا برآورد گردید و بالاترین میانگین مربوط به بعد شرایط فیزیکی و محیط کار بود. آنالیز آماری نشان داد که نوع شغل، سابقۀ کار و رضایت از شغل ارتباط معناداري با استرس شغلی دارند (0001/0P =). نتیجه‌گیری: به نظر می‌رسد کارکنان آتش‌نشانی در معرض خطر اختلال استرس شغلی قرار دارند و نوع شغل و سابقۀ کار عوامل اصلی موثر بر آن می‌باشند. استرس شغلی نیز می‌تواند موجب کاهش رضایت شغلی ‌گردد.

Title: بررسی غلظت سرب در ناخن افراد به عنوان نشانگر زیستی

http://hsr.mui.ac.ir/index.php/jhsr/article/view/2168

مقدمه: اصفهان به لحاظ داشتن صنایع متعدد و ترافیک سنگین یکی از آلوده‌ترین شهرهای ایران می‌باشد. این تحقیق به منظور استفاده از ناخن به عنوان یک نشانگر زیستی جهت بررسی اثر آلودگی هوا بر شهروندان اصفهانی در مقایسه با شهروندان چادگانی انجام شد. روش‌ها: دو گروه 65 نفری در محدوده سنی 5 تا 50 سال شامل زنان و مردان در هر دو شهر انتخاب و حدود 1 گرم از ناخن هر یک از آن‌ها جمع‌آوری شد. نمونه‌ها پس از آماده سازی و هضم برای تعیین غلظت سرب توسط دستگاه جذب اتمی مورد آنالیز قرار گرفتند. یافته‌ها: آزمون آنالیز واریانس بر روی اطلاعات مربوط به سن، جنس ، شغل و مواجهه با سیگار در افراد انتخاب شده در دو شهر نشان داد که جمعیت انتخاب شده در دو شهر کاملاً یک‌دست و مشابه می‌باشند و اختلاف معنی داری بین غلظت سرب در ناخن افراد مورد مطالعه در دو شهر وجود دارد (001/0 ≥ P). میانگین غلظت سرب در ناخن ساکنین اصفهان و چادگان به ترتیب برابر 1037/0 و 0875/0 میلی گرم بر گرم به‌دست آمد. نتیجه‌گیری: با توجه به مشابه بودن جمعیت مورد مطالعه در دو شهر و عدم وجود اختلاف معنی‌دار بین میانگین غلظت سرب در ناخن با نوع شغل، جنس و مواجهه با سیگار در افراد مورد مطالعه، مشخص می‌شود که اختلاف معنی‌دار موجود بین میانگین غلظت سرب در ناخن افراد مورد مطالعه ساکن اصفهان و چادگان (001/0 ≥ P) مربوط به محل سکونت آن‌ها می‌باشد و بنابراین مواجه با آلاینده‌های هوا در شهر اصفهان بر روی غلظت سرب در ناخن افراد مورد مطالعه ساکن اصفهان مؤثر بوده است. بنابراین استفاده از ناخن به عنوان یک نشانگر بیولوژیک برای بررسی غلظت سرب ناشی از آلودگی هوا در شهر اصفهان مناسب می‌باشد.

Title: مدل‎های شکنندگی توام برای مدل‎بندی همزمان پیشامدهای بازگشتی و یک رخداد نهایی با استفاده از روش برآوردیابی ماکزیمم درستنمایی تاوانیده و کاربرد آن در داده‌های پزشکی

http://hsr.mui.ac.ir/index.php/jhsr/article/view/2407

مقدمه: اطلاعات مربوط به پیشامدهای بازگشتی در مطالعات گذشته‎نگر می‎تواند با رها کردن دوره پیگیری، به پایان رسیدن دوره مطالعه و یا رخداد یک پیشامد نهایی مانند مرگ و یا ابتلا به یک بیماری با وضعیت ثابت، به پایان برسد. در این صورت پیشامد نهایی اصلی با پیشامدهای بازگشتی گذشته وابسته می‎شود و فرض سانسور شدن نا‎آگاهی‎بخش پیشامدهای بازگشتی بوسیله رخداد نهایی که فرض بسیاری از تحلیل‎های آماری است، نقص می‎شود. مدل‎بندی توام راه حلی است که در چنین وضعیتی به دلیل قابلیت مطالعه هم‎زمان دو فرآیند و دستیابی به برآوردهای نااریب و کافی برای پارامترها، جهت تحلیل بسیاری از مطالعات پیگیری مناسب است. روش‌ها: در این مطالعه مدل شکنندگی توام با روش برآوردیابی ماکزیمم درستنمایی تاوانیده مورد بررسی قرار گرفته و طی یک مثال بالینی کاربرد این نوع مدل‎بندی توأم در بررسی عوامل مؤثر بر تکرار وضعیت پره‌دیابت (پیشامد بازگشتی) و ابتلای نهایی فامیل افراد دیابتی به دیابت نوع دوم (رخداد نهایی) در یک مطالعه هم‎گروهی از بستگان درجه اول بیماران مبتلا به دیابت نوع دو، مراجعه کننده به مرکز تحقیقات غدد درون‎ریز و متابولیسم اصفهان نشان داده شده است. تحلیل‎ها با بکارگیری نرم افزار2.15.2R- صورت گرفته است. یافته‌ها: خطر بازگشت پره‎دیابت با تعدیل اثر انرژی دریافتی در افراد با مصرف کربوهیدرات بالای 65 درصد انرژی دریافتی 48/3 برابر بیشتر از مصرف کربوهیدرات کمتر از 40 درصد انرژی می‎باشد. خطر ابتلا به واقعه نهایی دیابت با تعدیل اثر انرژی دریافتی در افراد با مصرف پروتئین بین 12 تا 15 درصد انرژی دریافتی، 19/0 برابر بیشتر از مصرف پروتئین کمتر از 12 درصد انرژی دریافتی و مصرف پروتئین بالای 15 درصد انرژی دریافتی، 19/0 برابر بیشتر از مصرف پروتئین کمتر از 12 درصد انرژی دریافتی می‎باشد. نتیجه‌گیری: تغییر روش زندگی بر حسب اصلاح عملکرد تغذیه‌ای و بکارگیری روشی مبنی بر تعدیل شاخص‌های تن‎سنجی می‌تواند به‎عنوان عواملی مؤثر بر کاهش عملکرد ژنتیکی در ابتلا به بیماری دیابت نوع دو مورد استفاده قرار گیرد.
